# Flower-Visiting Insect Diversity Within Buckwheat Crops: An Underutilized Crop for Sustainable Economic Livelihoods

**DOI:** 10.3390/insects17020200

**Published:** 2026-02-13

**Authors:** Kedar Devkota, Prashant Rijal, Charles Fernando dos Santos

**Affiliations:** 1 Faculty of Agriculture, Agricultural and Forestry University, Chitwan 13712, Nepal; 2 Institute of Agriculture and Animal Science, Tribhuwan University, Kathmandu 44600, Nepal; aprashantrijal@gmail.com; 3 Departamento de Fitossanidade, Faculdade de Agronomia, Universidade Federal do Rio Grande do Sul, Porto Alegre 91540-000, Brazil

**Keywords:** agrosystems, economic, pollination, nutritional security, yield

## Abstract

Buckwheat (*Fagopyrum esculentum*) is an important source of nutrition for humans, providing essential nutrients such as protein, fiber, vitamins, and minerals. Its cultivation is highly attractive to flower-visiting insects, which find abundant nectar and a moderate amount of pollen grains. This study aimed to characterize the taxonomic diversity and composition of flower-visiting insect communities in buckwheat crops across two sites in Chitwan district, Nepal and to assess whether temperature and relative humidity influence community structure. We further quantified the contribution of insect pollination to buckwheat yield by comparing pollinator-excluded plots (net-covered) with open-pollinated plots. In addition, we estimated the economic value of insect-mediated pollination and the nutritional contribution of buckwheat production on a per capita basis. Data were analyzed using non-metric multidimensional scaling, permutational multivariate analysis of variance, similarity percentage analysis, and (generalized) linear mixed-effects models. We found significant differences in flower-visiting insect community composition between the two study sites, independent of temperature and relative humidity, with twelve taxa contributing most to this dissimilarity. Open-pollinated plots exhibited higher buckwheat yields than pollinator-excluded plots, highlighting the importance of insect visitation for crop production. Despite the presence of managed *Apis* species, we recorded frequent visitation by flies and solitary bees, indicating that these taxa are likely important contributors to buckwheat pollination at local scales. Similarly, insect-mediated pollination significantly increased buckwheat production, and its absence would result in substantial economic losses of USD 2.6 million and reduced nutritional contributions, highlighting the vulnerability of buckwheat-based food security for the Nepalese communities due to pollinator decline.

## Simple Summary

This study was carried out on Buckwheat, a pseudocereal crop, to examine the diversity of flower-visiting insects and their effects on the crop production at the two commercial sites of Chitwan District, Nepal. Overall, we found the 24 insect taxa and noted distinct variations in insect assemblages between sites, suggesting that regional ecological and landscape factors play a significant role. Over 70% of the buckwheat yield was due to the insect pollination. Although managed honey bees were present, frequent visits by flies and solitary bees indicate that non-*Apis* taxa make significant contributions to buckwheat pollination at local scales. The significant economic and nutritional benefits were brought to rural communities by insect-mediated pollination, which increased the availability of vital micronutrients for farming householdsand contributed about USD 2.6 million in production value. Our findings show that the decline in pollinators will cause substantial yield reductions, pose threats to the economy and food security through production losses, and reduce the nutritional value of agricultural products. These results highlight how necessary it is to implement conservation and management strategies that will aid both managed and wild insect pollinator species in and around agricultural production landscapes.

## 1. Introduction

Crop production is a cornerstone of agricultural systems, underpinning both the economic stability and nutritional security of a growing global population [1,2]. Its importance has become increasingly evident in recent decades as agriculture faces mounting pressures from climate change and rising food insecurity [3,4]. Under these conditions, animal-mediated pollination represents a critical ecosystem service, supporting agricultural productivity and helping mitigate nutritional deficiencies, particularly in smallholder farming systems [5,6].

Agricultural crop systems worldwide rely on a diverse assemblage of insect pollinators, with animal-mediated pollination contributing to approximately 35% of global food production [7]. Insect pollination enhances crop yields without the need for continuous external inputs, thereby supporting farmer income and rural livelihoods. This essential ecosystem service is estimated to contribute between USD 235 and 577 billion annually to the global agricultural economy [8]. Demand for pollination services has increased in recent decades due to the expanding cultivation of pollinator-dependent crops [9,10]. However, widespread declines in pollinator populations driven by habitat loss, pesticide exposure, pathogens, and other anthropogenic pressures raise serious concerns regarding the long-term sustainability of agricultural production and global food security [8,11,12,13].

Buckwheat (*Fagopyrum esculentum* Moench) is cultivated in many parts of the world, with a current global production of approximately 2.2 million tons [14]. In Nepal, it is grown primarily on marginal lands across 61 of the country’s 75 districts, spanning elevations from 60 to 4500 m, and particularly prevalent in hilly and mountainous regions [15]. Often referred to as a ‘poor man’s cereal’, buckwheat is nutritionally valuable, being rich in protein, dietary fiber, vitamins, and minerals [16,17]. Its gluten-free properties make it an important dietary alternative for individuals with celiac disease or gluten intolerance [18,19].

Insect pollination is essential for buckwheat production due to its self-incompatibility and distylous floral morphology (pin and thrum flowers), which necessitate pollen transfer between complementary flower types. Buckwheat flowers attract a diverse assemblage of visitors, including bees, flies, butterflies, and moths [20]. Depending on whether insects visit pin or thrum flowers, pollen adheres to different parts of their bodies, increasing the likelihood of successful transfer to the stigma of the opposite morph during subsequent visits [21,22].

The diversity of insect taxa contributing to buckwheat pollination supports both income generation and nutritional security among marginalized and smallholder farmers [23]. Accordingly, understanding the composition and dynamics of insect communities associated with buckwheat during the flowering period is essential for informing conservation and management strategies that promote pollinator diversity, identify key pollinating taxa, and maintain ecosystem functioning. Although buckwheat cultivation in Chitwan District, in Nepal, occurs within a relatively narrow climatic range, local agroecological and landscape-level differences may influence the composition and structure of flower-visiting insect communities. Comparing pollinator assemblages between Fulbari and Meghauli, two major buckwheat-producing areas, allows us to assess whether pollination services are consistent across sites or shaped by local ecological contexts. Such comparisons are essential to understand how spatial variability in pollinator diversity may translate into differences in crop productivity, economic value, and nutritional contributions.

Accordingly, this study aimed to evaluate the diversity and composition of flower-visiting insect communities contributing to buckwheat yield in the Chitwan, District of Nepal [24]. Specifically, we sought to (i) characterize the diversity and composition of flower-visiting insects associated with buckwheat cultivation, (ii) determine whether pollinator community structure differs between the two major production sites, Fulbari and Meghauli, and (iii) quantify the contribution of insect pollination to buckwheat yield, as well as its economic value and nutritional supply.

## 2. Materials and Methods

### 2.1. Study Sites

The study was conducted in the Chitwan, District of Nepal (27°35′ N, 84°30′ E), which lies at elevations ranging from approximately 150 to 350 m above sea level, depending on location. The district has a mean annual temperature of about 24.4 °C and receives an average annual precipitation of approximately 1500 mm, with most rainfall occurring during the monsoon season. Chitwan is one of Nepal’s most productive agricultural regions, characterized by fertile soils and diverse agro-climatic conditions that support traditional farming systems, including rice, wheat, maize, mustard, and buckwheat cultivation.

Buckwheat is cultivated in Chitwan between November and February and relies on insect-mediated pollination for optimal yields. Accordingly, we selected two major buckwheat-producing sites within the district: Meghauli (27°34′49″ N, 84°13′36″ E; 150 m a.s.l.) and Fulbari (27°38′27.27″ N, 84°22′11.92″ E; 150 m a.s.l.), which are located approximately 15 km apart. Together, these sites account for nearly 80% of the district’s buckwheat production.

A total of eight buckwheat fields were selected as research sites, with four fields in each location. Within each field, a study area measuring 50 m × 25 m was established, characterized by homogeneous and continuous crop cover. This experimental design followed globally recommended protocols for pollination studies [25]. Although Fulbari and Meghauli share similar climatic conditions, they differ in surrounding land-use composition, degree of agricultural intensification, and proximity to semi-natural habitats, factors known to influence pollinator community structure and availability.

### 2.2. Insect Sampling

To sample a diverse assemblage of flower-visiting insects, we employed pan traps, a widely used passive sampling method that effectively attracts insects by mimicking the visual cues of floral resources [26,27]. Pan traps are easy to use, lack collector bias, and often capture a greater diversity of potential pollinators. However, it may escape the large number of insects, especially bee species and measure insect availability rather than actual flower visitation or pollination [28]. Traps were deployed along a 150 m transect within each buckwheat field. Pan traps of three colors (yellow, blue, and white) were arranged sequentially along the transect, with three traps randomly positioned within each 25 m segment and a minimum spacing of 5 m between traps [29].

Following the protocol recommended by the Canadian Pollinator Initiative, we deployed pan traps during the buckwheat flowering period. At each field, traps were arranged along transects in six sampling rounds, with each round consisting of 18 pan traps; six per color (yellow, blue, and white). In total, 144 pan traps (18 traps × 8 fields) were deployed across the study sites and operated on four sampling days to encompass the full flowering period of buckwheat. Traps were set in the fields from 09:00 to 17:00 h.

Insects captured in the pan traps were pinned and identified to genus or species level whenever possible. Voucher specimens were deposited in the entomological collection of the Agriculture and Forestry University, Rampur, Chitwan, Nepal. In parallel, daily environmental variables, including minimum and maximum temperature, precipitation, and relative humidity, were recorded in each of the eight fields using a hygrometer.

### 2.3. Insect Pollination Effects on Buckwheat Yield

To assess the effect of insect communities on buckwheat yield during the flowering period, we conducted a pollination experiment comprising four experimental blocks (2 × 2 m) at each study site. Within each block, two treatments were established prior to flowering: (i) pollination access plots (1 × 1 m), which allowed unrestricted visitation by flower-visiting insects, and (ii) pollination exclusion plots (1 × 1 m), which were covered with nylon mosquito nets to prevent insect visitation. Differences in yield between these treatments, referred to as inside net and outside net yield, were used as a proxy to estimate the contribution of insect-mediated pollination to buckwheat production.

Within each treatment, five plants per plot were randomly selected and tagged to quantify the mean number of flowers, fruit set rate, and mean seed weight. The experimental unit was the plot, with the five tagged plants treated as subsamples used to estimate plot-level means for each treatment. Although exclusion nets may alter local microclimatic conditions, both treatments were established within the same fields and sampling period, minimizing systematic microclimate bias between treatments. After flowering ceased, nylon nets were removed in pollination exclusion plots to allow tagged plants to mature under field conditions until harvest. At harvest, the number of seeds per plant was recorded for each plot, and total seed weight per plant was measured using a precision scale. These yield components were used as key proxies of buckwheat productivity and to assess the dependence on insect-mediated pollination of the crops.

### 2.4. Pollination Contribution on Income, Production and Nutritional Value from Buckwheat

To quantify the contribution of insect pollination to buckwheat yield, the percentage increase in seed yield under pollination access relative to pollination exclusion was calculated using the following formula:

(1) $$Pollinator\;dependency\left(\%\right)=\frac{Yield\;in\;Pollination\;Access-Yield\;in\;Pollination\;Exclusion}{Yield\;in\;Pollination\;access}\times 100$$

Pollinator dependence estimates were used to calculate the production value of insect pollination (PVIP) for buckwheat in Nepal, based on national production data obtained from FAO datasets spanning 2002–2023. In addition, the nutritional contribution of total buckwheat production was estimated on a per capita annual basis using nutritional composition data from the USDA. Per capita buckwheat consumption for the year 2023 was calculated using the formula in Equation (2), assuming all nationally produced buckwheat was available domestically for human consumption. The resulting values reflect the potential nutritional contribution attributable to insect pollination, without adjusting for post-harvest losses, retained seed, processing losses, or international trade.

(2) $$Per\;capita\;consumption=\frac{Total\;yield\;of\;Buckwheat}{Total\;Population}$$

PVIP estimates represent a first-order scenario-based extrapolation assuming constant average market prices and proportional scaling of pollination dependence, and are intended to illustrate the potential magnitude of pollination services rather than provide precise economic forecasts. As such, long-term price variability and uncertainty ranges were not explicitly modeled, PVIP values should be interpreted as conservative approximations.

### 2.5. Data Analysis

#### 2.5.1. Insect Diversity Comparison Among Sites

Data analyses were conducted using the statistical programming language R [30,31]. The sampling unit for all multivariate analyses was an individual sampling event, defined as a single standardized flower-visitation survey conducted at a given site on a given sampling day, with each sampling event treated as an independent replicate. Surveys were conducted across multiple sampling days at each site, and data were not pooled across days prior to analysis in order to preserve temporal replication and within-site variability. To examine patterns of structure and dissimilarity in flower-visiting insect communities between the two study sites, we applied non-metric multidimensional scaling (NMDS). To reduce potential biases arising from the numerical dominance of particular taxa, the species abundance matrix was standardized using the Hellinger transformation implemented via the “decostand” function in the R package vegan [32]. This transformation is widely recommended for community-level ordination of species abundance data, as it reduces the influence of highly abundant taxa while retaining information on relative species composition. The transformed data were subsequently used to construct a Bray–Curtis dissimilarity matrix with the “vegdist” function. The Bray–Curtis index was selected because it is well suited for ecological count data, is robust to joint absences, and emphasizes relative differences in community composition among samples. The Hellinger transformation minimizes the influence of highly abundant taxa through square-root normalization, while the Bray–Curtis index quantifies community dissimilarity based on relative differences in taxonomic composition and abundance between sites.

NMDS ordination was performed using the “metaMDS” function with three dimensions (k = 3), excluding zero dissimilarities. The resulting stress value (<0.20) indicated an adequate fit of the ordination model. To facilitate graphical interpretation, contour surfaces were overlaid onto the NMDS ordination to examine potential relationships between insect community structure and two environmental variables: temperature and relative humidity. These relationships were visualized using thin-plate splines generated through generalized additive models (GAMs) implemented with the “ordisurf” function in the vegan package [32].

In addition, the potential influence of temperature and relative humidity on community ordination was assessed by fitting environmental vectors using the “envfit” function. Thin-plate spline surfaces provide a smooth interpolation of environmental gradients across ordination space, whereas the envfit analysis evaluates the statistical significance and directional strength of environmental variables in structuring taxa distributions within the NMDS framework.

Differences in pollinator community structure between Fulbari and Meghauli were evaluated using permutational multivariate analysis of variance (PERMANOVA) with 1999 permutations, implemented via the “adonis2” function in the vegan package. Because PERMANOVA can be sensitive to differences in multivariate dispersion among groups, we explicitly assessed the homogeneity of group dispersions prior to interpretation of PERMANOVA results. This analysis quantifies the average distance of samples to their group centroids, allowing comparison of dispersion patterns between the two sites, with significance assessed using permutation tests. In addition, similarity percentage (SIMPER) analysis was conducted using the ‘simper’ function as a descriptive tool to identify the taxa contributing most to the observed dissimilarities in insect community composition.

#### 2.5.2. Buckwheat Yield Comparison Among the Treatments

To evaluate the effects of flower-visiting insects on buckwheat productivity, we fitted two linear mixed-effects models (LMMs) and one generalized linear mixed-effects model (GLMM) to analyze yield (g), 1000-seed weight, and total seed number, respectively. The GLMM was specified with a Poisson error distribution and fitted using the “glmer” function in the lme4 package [33]. In all models, the response variables corresponded to the yield components described above, while pollination treatment (pollinator exclusion vs. pollination access) was included as a fixed effect. To account for potential spatial variability and non-independence among observations, study sites were included as random effects in all models. This modeling structure allowed variation among sites to be explicitly accounted for, thereby improving model inference and generalizability.

## 3. Results

### 3.1. Flower Visiting Insects Diversity

In general, we found a total of 26 insect taxa, categorized into different taxonomic groups. The bees (13 taxa) include *Apis mellifera* (Western honeybee), *Apis cerana* (Asian honeybee), *Apis dorsata* (giant honeybee), *Apis florea* (dwarf honeybee), *Halictus* spp. (sweat bees), *Andrena* spp. (mining bees), *Megachile* spp. (leafcutter bees), *Colletes* spp. (plasterer bees), *Osmia* spp. (mason bees), *Lasioglossum* spp. (small sweat bees), *Bombus* spp. (bumblebees), *Xylocopa* spp. (carpenter bees), and *Ceratina* spp. (small carpenter bees). The wasps (6 taxa) consisted of *Vespa* spp. (hornets), adults of Ichneumonidae (ichneumon wasps), *Polistes* spp. (paper wasps), *Eumenes maxillosus* (potter wasp), *Evania appendigaster* (blue-eyed ensign wasp), and an unidentified genus referred to as a wasp. The flies (3 taxa) included *Musca* spp. (house flies), *Eristalis cerealis* (hoverfly), and adults of Sarcophagidae (flesh flies), while, the sawflies were assigned to a single taxon, *Athalia lugens* (turnip sawfly). Lastly, the butterflies (3 taxa) were represented by *Pieris brassicae* (cabbage butterfly), *Junonia almana* (peacock pansy), and *Danaus chrysippus* (plain tiger).

**Figure 1:**
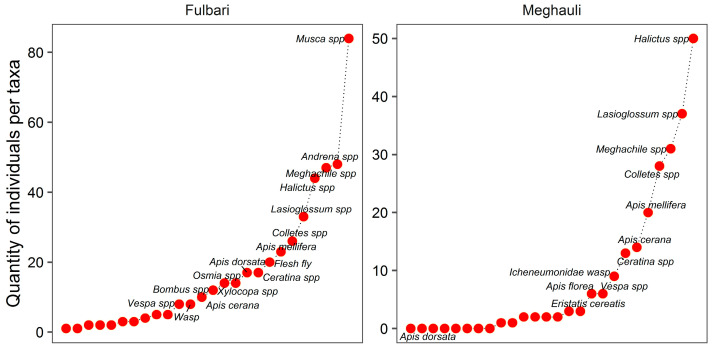
Rank–abundance (Whittaker) plots illustrating the abundance and relative dominance of flower-visiting insect taxa in buckwheat crops at the Fulbari and Meghauli sites in Nepal. The *y*-axis represents the number of individuals sampled, with each taxon labeled next to its corresponding data point. This plot is inspired by the Whittaker plot, as it visually represents the relative similarity and differences in insect community composition between the two sites. Overall, the plot suggests that Fulbari showed a greater diversity of insect taxa compared to Meghauli, indicating potential differences in wild and managed insect availability and ecosystem dynamics between the regions.

A total of 683 insect individuals were recorded from pan trap samples across the eight study fields in Fulbari and Meghauli, with 453 individuals collected in Fulbari and 230 in Meghauli (Figure 1). Community structure was visualized using rank–abundance (Whittaker) plots, in which taxa are ordered along the x-axis by increasing abundance and the y-axis represents the number of individuals per taxon. This approach facilitates comparison of species composition, dominance patterns, and evenness between sites.

The slope of the rank–abundance curve provides insight into community structure, with steeper slopes indicating dominance by a few taxa and lower evenness, and shallower slopes reflecting more even distributions among taxa. In Fulbari, *Musca* spp. was the most dominant taxon, followed by wild and solitary bees such as *Andrena* spp. and *Megachile* spp. (Figure 1, left). In contrast, the Meghauli community was dominated by wild and solitary bees, particularly *Halictus* spp., followed by *Lasioglossum* spp. and *Megachile* spp. (Figure 1, right). Overall, both sites exhibited long upper tails in their rank–abundance distributions, indicating the presence of numerous taxa with low relative abundances.

**Figure 2:**
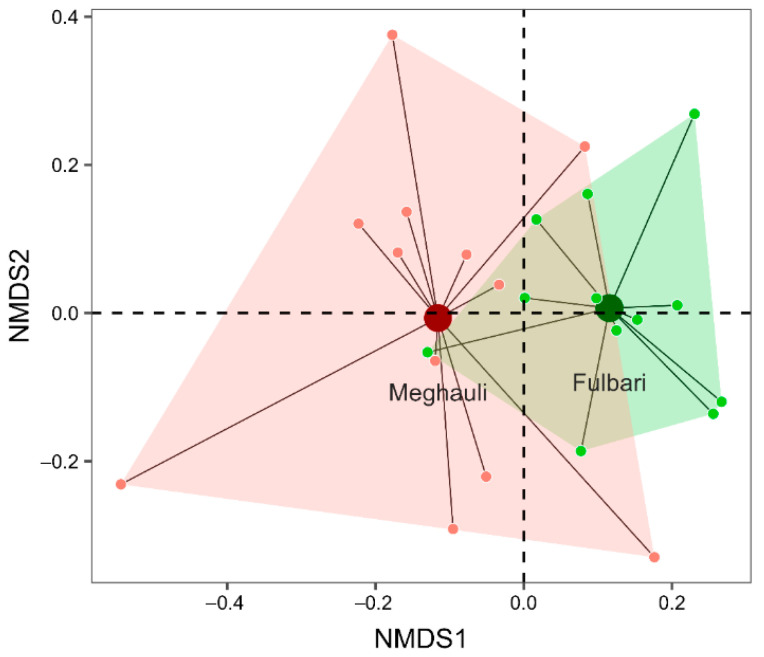
Non-parametric multidimensional scaling (NMDS) plots exhibiting the insect community composition in buckwheat-cultivating sites, Fulbari and Meghauli, Nepal, depicting their structural differences in terms of beneficial insect diversity. Here, we can see how insect communities were organized within each site, including variations in taxa composition and potential ecological influences shaping pollinator distribution in buckwheat fields. Points represent individual sampling units, colored by site (Meghauli: red; Fulbari: green). Large filled circles indicate site centroids, and connecting lines show the distance of each sample to its respective centroid (within-site dispersion). Shaded polygons represent the convex hull enclosing samples from each site, illustrating the extent of community variation.

To evaluate whether flower-visiting insect communities differed between the two major buckwheat-producing sites, we used NMDS to compare pollinator assemblages between Fulbari and Meghauli. Ordination analysis of insect community composition (stress = 0.16; non-metric fit, R^2^ = 0.97) revealed a significant difference in community structure between Fulbari and Meghauli (PERMANOVA: F_(1, 22)_ = 2.68, R^2^ = 0.10, *p* = 0.001; Figure 2). These results indicate that insect assemblages at the two sites were compositionally distinct. In contrast, analysis of multivariate dispersion showed no significant difference in within-group variability (betadisper: F_(1, 22)_ = 3.63, *p* = 0.072), suggesting that although community composition differed between sites, both exhibited comparable levels of internal heterogeneity.

**Figure 3:**
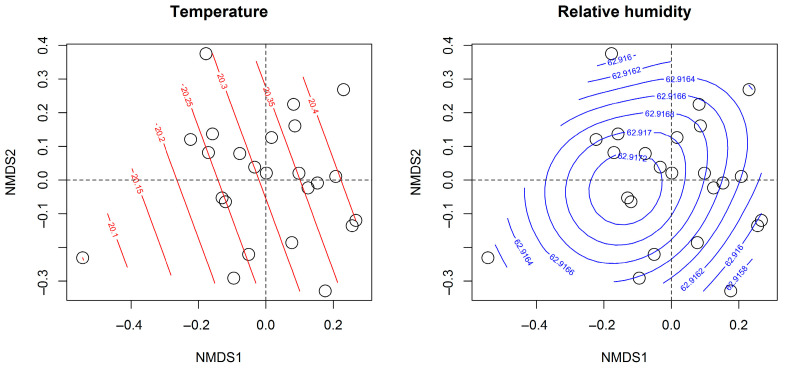
Ordisurf plots showing the relationship between insect community composition and weather variables in buckwheat fields. The left panel represents temperature gradients, while the right panel depicts relative humidity, both modeled using generalized additive models (GAMs) in R. Site samples are represented by circles, and contour lines indicate areas of higher and lower values for each variable. These plots provide insights into how temperature and relative humidity influence insect community composition. As a whole, they help to identify potential environmental drivers shaping pollinator distribution across study sites.

Despite the observed differences in insect community composition, neither temperature (R^2^ = 0.10, *p* = 0.31) nor relative humidity (R^2^ = 0.06, *p* = 0.49) showed a significant influence on taxa ordination (Figure 3), suggesting that other ecological or landscape-level factors may underlie the observed community differentiation. Although site identity explained a modest proportion of community variation (R^2^ = 0.10), differences in surrounding land use and habitat structure are proposed as plausible, but untested, hypotheses rather than demonstrated mechanisms. Because landscape variables were not explicitly quantified, causal attribution to land-use effects should be interpreted with caution. Furthermore, SIMPER analysis identified 12 of the 26 recorded taxa as major contributors to the dissimilarity between Fulbari and Meghauli (Table 1), highlighting specific insect groups that drive the structural divergence between the two sites.

### 3.2. Crop Productivity and the Role of Flower-Visiting Insects

**Figure 4:**
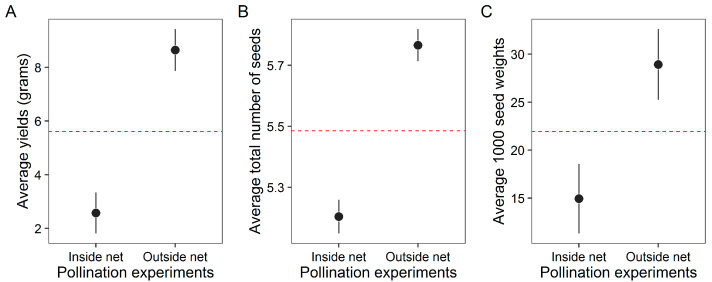
Expected effects of pollinating insects on buckwheat crops in Nepal. (**A**) Average yield per plant (g); (**B**) Average total number of seeds per plant; (**C**) Average weight of 1000 seeds. The points represent average values, while the vertical lines indicate 95% confidence intervals. Dashed horizontal lines (red color) denote the overall mean value, included to enhance visualization and comparison between experiments. These results illustrate the potential impact of flower-visiting insects, assigned here as potential pollinators on buckwheat productivity.

Buckwheat yield was significantly higher in pollination access plots, here referred to as outside net yield, than in pollinator exclusion plots, here referred to as inside net yield, with increases observed in total yield (LMM: χ^2^ = 148, df = 1, *p* < 0.001) and 1000-seed weight (LMM: χ^2^ = 44, df = 1, *p* < 0.001; Figure 4A,B). In addition, total seed number was substantially greater in open-pollinated plots (GLMM, Poisson family: χ^2^ = 1141, df = 1, *p* < 0.001; Figure 4C). Together, these results demonstrate that flower-visiting insect activity significantly enhances buckwheat productivity, as reflected by increased seed quantity and seed mass.

### 3.3. Economic and Nutritional Contribution of Pollination

**Figure 5:**
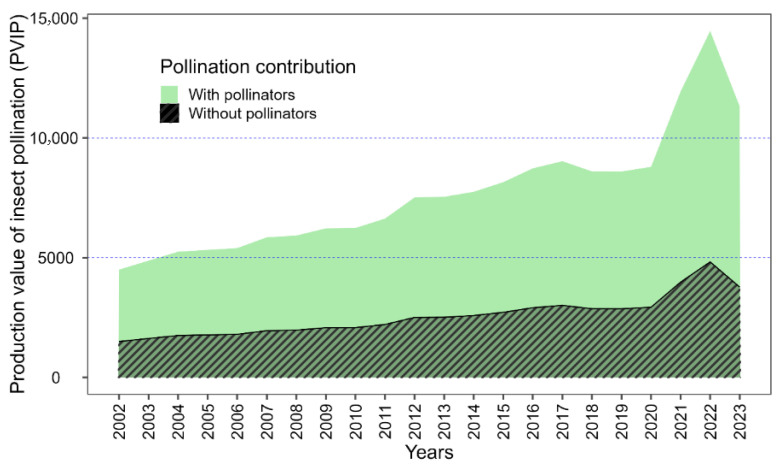
Quantifies the economic contribution of insect pollinators to buckwheat production over two decades. The solid green area depicts the actual production value supported by pollinators, while the black-and-white striped area illustrates the projected loss in production value in their absence. Years are displayed in annual increments along the x-axis, with production values (in appropriate units) on the y-axis. The visual contrast between the solid and striped areas emphasizes the role of pollinators in sustaining buckwheat productivity.

Figure 5 illustrates the strong dependence of buckwheat production on insect pollination, with production values under pollinator presence (solid green area) consistently exceeding the hypothetical scenario without pollinators (striped area). Field observations identified bees and flies as the dominant flower visitors, whose activity likely underpins the higher production value of insect pollination (PVIP), particularly during peak flowering periods. The magnitude of the striped area represents production losses in the absence of pollinators which highlights the economic vulnerability of buckwheat production to pollinator decline. Based on 2023 production estimates, the loss of pollination services would result in an economic loss of approximately USD 2.6 million to the Nepalese economy.

In addition to economic impacts, insect-mediated pollination substantially contributes to the nutritional supply. For 2023, the PVIP of buckwheat production was estimated to provide 2961.66 gm of carbohydrates, 140.83 gm of total fats, 546.77 gm of protein, as well as 4.14 g of thiamin, 16.57 g of riboflavin, 290 g of niacin, and 91.13 g of iron on a per capita basis, underscoring the nutritional importance of pollination services for food security.

## 4. Discussion

The differences in flower-visiting insect assemblages captured between Fulbari and Meghauli indicate spatial heterogeneity in the availability of potential pollinators within buckwheat fields, even within the same district. A total of 26 taxa were recorded, with more taxa of bees with huge variation in the flower-visiting insect abundance across the two sites. Such variation may plausibly be associated with differences in landscape structure, availability of semi-natural habitats, or local management practices, although these factors were not directly quantified in this study [34]. Given the strong pollinator dependence of buckwheat, spatial variation in pollinator assemblages may translate into differences in pollination efficiency and, ultimately, crop yield. Overall, our findings reveal the importance of conserving diverse pollinator communities across agricultural landscapes to stabilize buckwheat production and sustain its associated economic and nutritional benefits.

Understanding the drivers of insect community composition within buckwheat agroecosystems is essential for the conservation of flying insect biodiversity and the maintenance of key ecosystem functions [34,35,36]. Insects underpin multiple ecosystem services, including pollination, nutrient cycling, and biological pest regulation [37,38,39]. The marked differences in insect community composition observed between Fulbari and Meghauli, therefore, have important implications for the productivity and sustainability of buckwheat. These findings suggest that regional ecological dynamics may influence pollinator availability and, consequently, pollination efficiency and crop yields. Moreover, our results emphasize the need for targeted conservation and management strategies that promote wild insect diversity, thereby supporting long-term ecosystem stability and sustainable agricultural production, consistent with patterns reported for other pollinator-dependent cropping systems.

Given that site identity explained a modest proportion of community variation, and that within-group dispersion did not differ between sites, the observed differences are unlikely to be driven solely by the environmental variables examined in this study, namely temperature and relative humidity. Instead, unmeasured factors such as landscape composition, land-use intensity, soil characteristics, and local management practices may have contributed to the observed community differentiation. Thus, further research is needed to disentangle the relative influence of these drivers and to improve understanding of the ecological processes shaping pollinator community structure. Moreover, the distinct flower-visiting insect assemblages associated with buckwheat crops observed between Fulbari and Meghauli may reflect differences in the availability and diversity of wild insect populations, which could ultimately influence buckwheat productivity and quality, as reported for other pollinator-dependent agricultural systems [7,40,41,42].

Although this study primarily examined seed set and fruit set in relation to flower-visiting insect community composition, we hypothesized that the observed differences in insect assemblages between Fulbari and Meghauli have broader ecological implications, particularly for pollination services in buckwheat cultivation. Given the strong dependence of buckwheat on insect pollinators [43], spatial variation in pollinator community structure may directly affect pollination efficiency, reproductive success, and, ultimately, crop yield.

Beyond their role in crop pollination, the diversity and abundance of insect communities play critical role in broader ecosystem functioning and biodiversity conservation [34,44,45,46]. Insects in agricultural landscapes contribute to essential ecosystem processes such as nutrient cycling, biological pest regulation, and the maintenance of ecosystem stability. Consequently, declines in wild (non-managed) insect populations may result in cascading losses of ecosystem services and pose substantial threats to biodiversity, particularly in rural regions of Nepal and other landscapes where agricultural production is tightly coupled with surrounding natural ecosystems.

Our results showed that buckwheat is a highly pollinator-dependent crop, with yield differences exceeding 70% between the two experimental plots. Consequently, declines in pollinator populations lead to substantial production losses, ultimately reducing the economic income of rural farmers in Nepal [47]. Beyond these economic impacts, reduced buckwheat production may also diminish its nutritional availability to local populations. Although the estimated per capita nutritional contributions represent the potential supply derived from total national buckwheat production rather than actual dietary intake, they nonetheless indicate the scale at which pollinator decline could affect access to key macronutrients and micronutrients. Therefore, pollinator decline threatens not only farm livelihoods but also food and nutrition security, particularly in remote mountain and hill communities where buckwheat serves as a key staple and an important substitute for cereals [15,16,47]. Similarly, the production value of insect pollination represents the scenario-based production, which shows the potential magnitude of pollination-related economic losses rather than precise monetary forecasts [7,47]. The pronounced production gap between scenarios with and without pollinators is more serious in the context of pollination deficit in the major crops cultivated in Nepal [43] is consistent with global patterns observed in pollinator-dependent crops. The predominance of bee taxa, which are highly efficient pollen vectors, likely contributes to the elevated production value of insect pollination observed in this study. However, the projected production losses under pollinator absence indicate that current agricultural practices may be insufficient to sustain these insect populations. Accordingly, habitat enhancement measures (e.g., the establishment of wildflower strips) and reductions in pesticide use are proposed as precautionary strategies to conserve pollinator communities and mitigate risks to regional buckwheat production while also safeguarding the availability of nutritious food for farming communities. Future research should further examine how climate change may interact with landscape management to disrupt pollination services in this system.

## 5. Conclusions

In summary, our findings offer novel insights into the spatial variability of pollinator communities within a localized agricultural context, which can inform targeted strategies to improve buckwheat production, such as promoting habitat diversification through wildflower strips, reducing pesticide application, and integrating pollinator-friendly practices in marginal lands. By identifying key pollinating taxa and quantifying their contributions, this information empowers farmers and policymakers to enhance pollination efficiency, potentially stabilizing yields and mitigating risks from pollinator declines amid climate change and habitat loss.

Future studies should investigate unmeasured drivers such as landscape composition, soil health, and management practices to deepen the understanding of insect community dynamics. In addition, longitudinal experiments examining climate interactions and genetic diversity among buckwheat floral morphs may help identify adaptive pathways for developing resilient production systems in comparable agroecosystems worldwide.

## Figures and Tables

**Table 1:** Individual and cumulative contributions of key taxa differentiating insect communities in Fulbari (F) and Meghauli (M), Nepal. This analysis highlights the most influential insect taxa (collectively over 70% of the contribution) driving community differences between the two sites, presenting their individual contributions as well as their cumulative impact on overall community dissimilarity.

Taxa	Abundance		Individual Contribution	Cumulative Contribution
	Fulbari vs. Meghauli			
1. *Musca* spp.	84	0	0.13	0.13
2. *Megachile* spp.	47	31	0.06	0.19
3. *Lasioglossum* spp.	33	37	0.06	0.25
4. *Apis mellifera*	23	20	0.07	0.32
5. *Sarcophagidae*	20	2	0.06	0.38
6. *Halictus* spp.	44	50	0.06	0.44
7. *Apis dorsata*	17	0	0.05	0.49
8. *Apis cerana*	10	14	0.05	0.54
9. *Colletes* spp.	26	28	0.05	0.59
10. *Osmia* spp.	14	0	0.04	0.63
11. *Andrena* spp.	48	0	0.05	0.68
12. *Ceratina* spp.	17	13	0.04	0.72
			47% = average dissimilarity between both sites	

## Data Availability

The data that support the findings will be provided at ZENODO (https://doi.org/10.5281/zenodo.18621652, accessed on 1 February 2026) if this manuscript was accepted.
